# A Comparative Study in the Design of TiO_2_ Assisted Photocatalytic Coatings Monitored by Controlling Hydrophilic Behavior and Rhodamine B Degradation

**DOI:** 10.3390/ma16072589

**Published:** 2023-03-24

**Authors:** Xabier Sandua, Pedro J. Rivero, Ana Conde, Joseba Esparza, Rafael Rodríguez

**Affiliations:** 1 Engineering Department, Public University of Navarre, Campus Arrosadía s/n, 31006 Pamplona, Spain; 2 Institute for Advanced Materials and Mathematics (INAMAT2), Public University of Navarre, Campus Arrosadía s/n, 31006 Pamplona, Spain; 3 National Center for Metallurgical Research (CENIM-CSIC), Gregorio del Amo Avenue 8, 28040 Madrid, Spain; 4 Centre of Advanced Surface Engineering, AIN, 31191 Cordovilla, Spain

**Keywords:** photocatalysis, wettability, electrospraying, spraying, dip coating

## Abstract

This work presents a comparative study related to the photocatalytic efficiency associated with wettability measurements and organic dye degradation, as well as other relevant properties (i.e., corrosion resistance, roughness, wettability, and adhesion to a substrate). The photocatalytic precursors are titanium dioxide nanoparticles (TiO_2_ NPs) which are dispersed onto a polymeric electrospun fiber matrix by using three different deposition techniques such as electrospraying, spraying, and dip-coating, respectively. In this work, the host electrospun matrix is composed of poly(acrylic acid) fibers crosslinked with cyclodextrin (β-CD), which shows a good chemical affinity and stability with the other deposition techniques which are responsible for incorporating the TiO_2_ NPs. In order to evaluate the efficacy of each coating, the resultant photocatalytic activity has been monitored by two different tests. Firstly, the reduction in the water contact angle is appreciated, and secondly, the degradation of an organic dye (Rhodamine B) is observed under UV irradiation. In addition, the final roughness, adherence, and pitting corrosion potential have also been controlled in order to determine which solution provides the best combination of properties. Finally, the experimental results clearly indicate that the presence of TiO_2_ NPs deposited by the three techniques is enough to induce a super hydrophilic behavior after UV irradiation. However, there are notable differences in photocatalytic efficiency on the Rhodamine B as a function of the selected deposition technique.

## 1. Introduction

Sustainable and environmentally friendly methods are increasingly required nowadays for organic pollutant treatments. The photocatalysis phenomenon is one of the best examples of how to clean air [1,2,3] and water [4,5,6] in a green way [7]. Furthermore, this photoinduced reaction is also presented in self-cleaning [8,9,10], antitumor activity [11,12,13], and self-sterilizing [14,15,16]. TiO_2_ is the most employed photocatalytic element due to its low cost, chemical stability, non-toxicity, and high reactivity [17]. This photochemical reaction occurs when TiO_2_ is irradiated with UV light, producing electron-hole pair on the surface of the semiconductor [18]. The creation of an electron hole gives rise to the formation of radicals that react with external molecules, inducing photocatalytic reactions [19]. One of the methodologies to evaluate the resultant photocatalytic efficiency is monitoring the degradation rate of organic dyes after UV light incidence [20]. An interesting approach to validate the photocatalytic behavior of the thin films doped with metallic oxide precursors is analyzing the change in the wettability. A clear example can be appreciated in [21], where the interaction of TiO_2_ with the induced UV light leads to a fast decrement of the water contact angle value, showing an intrinsic super hydrophilic surface because the water drop totally vanished. This fact is due to the formation of hydroxyl radicals during photocatalytic reactions. The stable hydroxyl radicals increase the surface energy of the sample, transforming the hydrophilic surface into a super hydrophilic one [22,23].

There are many phenomena that directly may affect the photocatalytic performance of the TiO_2_ particles, the most representative of the size, specific surface area, pore volume, pore structure, crystalline phase, and the exposed surface facets [24]. The main three phases of TiO_2_ semiconductor are anatase, rutile, and brookite, with anatase being the most active phase in terms of photocatalysis [25]. In addition, the separation and stability of the charges generated during photocatalysis reactions give rise to a reduction in charge recombination and, thus, an increase in photocatalytic activity [26]. The multiple morphologies (spherical, nanorods, fibers, tubes, sheets, or a combination) [27] are a key feature. However, the spherical shape is the most used due to its high specific surface area with a high pore volume and pore size.

It can be found in the bibliography several deposition techniques have been implemented in order to immobilize TiO_2_ particles with a wide variety of shapes and sizes, such as surface implantation via physical vapor deposition magnetron sputtering [28,29], sol-gel [30,31], Layer-by-Layer assembly [32,33], chemical vapor deposition [34,35], electrospraying [36,37], spraying [38,39], dip-coating [40,41], electrospinning [42,43] or atomic layer deposition [44,45]. Among all of them, this work is focused on the use of simple and low-cost surface treatments, which are easy to implement in a fast way. The first one is spraying, which is a simple liquid atomization process based on the application of a vacuum in a chamber while the solution is flowing along an atomizer nozzle with a certain pressure at room temperature [46,47]. This coating technique is a low-cost technology and allows the deposition of desired particles in a large surface area so that it can be easily applied in the industry [48]. Spraying permits one to obtain particles of different shapes and sizes, which can be interesting in several applications [49,50,51]. The second one is the electrospraying technique which is widely used for the construction of photocatalytic coatings [52,53]. This process is a type of liquid atomization process where a conductive solution flows through an electrically charged nozzle, generating droplets of the solution. Several parameters, such as flow rate, applied voltage, deposition distance, and solution conductivity, have to be perfectly controlled in order to obtain the desired coating [54]. In a similar way to the spraying process, electrospraying is used to immobilize spherical-shaped nanoparticles into the substrate. Nevertheless, electrospraying has several advantages compared to the spraying process, where smaller particle size can be obtained when a high electric voltage is applied in the tip of the syringe, reaching usual sizes values lower than 1 μm. Furthermore, a homogeneous distribution of particle size and a good dispersion of particles along the substrate is achieved, avoiding agglomerated compounds [55]. The third one is the dip-coating technique which is based on the immersion of the substrate in a solution where the functional particles are already presented, immobilizing those particles into the substrate. Dip-coating provides good purity and homogeneity features at room temperature, a nonhazardous process [56]. The particle immobilization capacity of dip-coating is greater than the previously explained methods and can even be adjusted to a wide range of substrate shapes [57]. Finally, electrospinning consists of the formation of fluid jets by the application of a high voltage in the tip of the syringe where the solution is pumped. Similar to electrospraying, parameters such as flow rate, applied voltage, deposition distance, and solution viscosity must be controlled [58,59]. This process allows the formation of porous coatings with a high functional area, and as low-cost technology, it is applicable at the industry or laboratory scales [60].

The main novelty of this work is the possibility of combining these previously described wet-chemistry techniques for the fabrication of highly efficient photocatalytic dry surfaces. According to this, the electrospinning process makes possible the formation of a functional electrospun fiber mat which acts as a host base matrix where different types of particles can be adhered [61,62]. In this sense, this electrospun fiber mat is employed as the base element for the immobilization of TiO_2_ by the further implementation of three different deposition techniques such as electrospraying, spraying, and dip coating, respectively. In this work, it is compared the combination of three different functional coatings such as electrospinning-electrospraying, electrospinning-spraying, and electrospinning-dip coating, in terms of photocatalytic activity. Finally, the corrosion has also been analyzed due to the possibility of applying these surface coatings onto components that are present in wastewater treatment plants. According to this, stainless steel, such as AISI 304, has been employed to simulate the materials which are used in pipelines, pumps, and tanks [63]. Previous works have demonstrated that TiO_2_ particles deposited in the outer layer of the film act as a barrier to electrolytes, improving the corrosion resistance of the thin film [64,65]. This study works with a weak polyelectrolyte with intrinsic hydrophilic behavior such as poly(acrylic acid) (PAA), which retrogresses the corrosion properties of the substrate, forming an aggressive electrolyte media between substrate and coating, although the experimental results corroborate that the presence of TiO_2_ particles over the coating will maintain the initial electrochemical properties of the substrate.

## 2. Materials and Methods

### 2.1. Materials

The electrospinning solution is composed of a mixture of poly(acrylic acid) (PAA; Mw ≈ 450,000) and β-cyclodextrin (β-CD, purity 98%), which were diluted in ethanol (99%). The solution used in the three types of depositions techniques (electrospraying, spraying, and dip-coating) was made by titanium oxide (TiO_2_; pure anatase nanopowder < 25 nm), diluted in ultrapure water. Then, cetyltrimethylammonium bromide (CTAB; purity 98%) and polyethylene oxide (PEO; Mw ≈ 400,000) were added to the solution. As an organic dye for the photocatalytic tests, Rhodamine B (RhB; purity 95%) has been employed. All the chemicals were obtained from Sigma-Aldrich (St. Louis, MO, USA). All reagents were used without any further purification, and ultrapure water with a resistivity of 18.2 MΩ·cm was employed as deionized water for the preparation of the solutions.

The functional coatings have been implemented onto standard glass slides (75 mm × 50 mm) in order to analyze the morphological surface (thickness, roughness, and microscopy) and photocatalytic tests. Finally, adhesion and electrochemical analysis were performed on austenitic stainless steel substrates (AISI 304) with a dimension of 80 × 60 mm^2^.

### 2.2. Deposition Techniques

**Figure 1:**
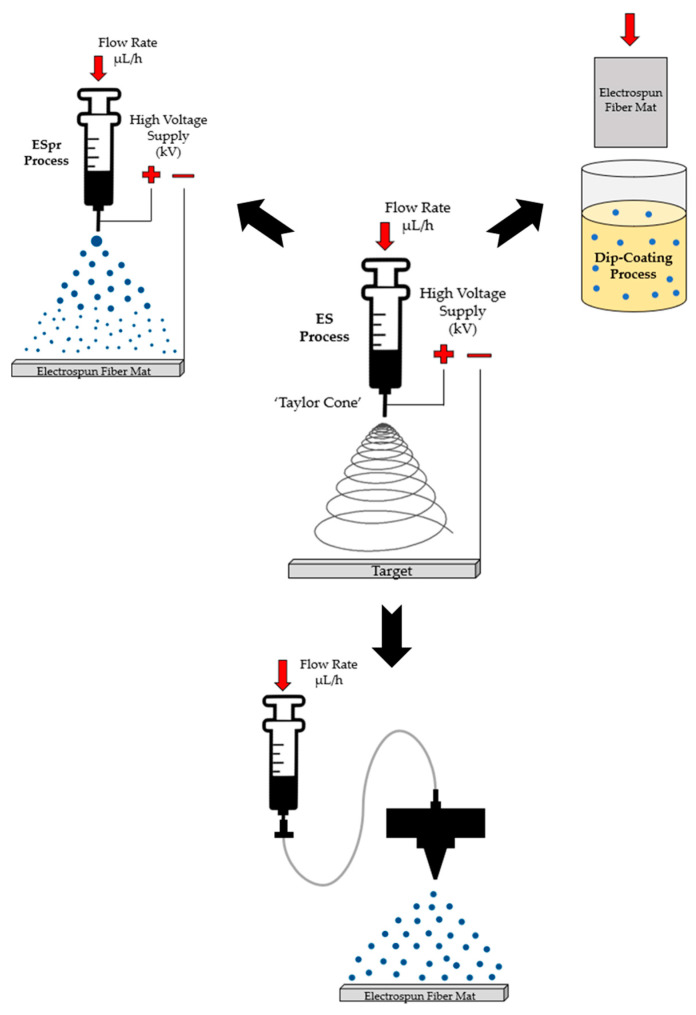
Schematic representation of the deposition techniques. Starting with the deposition of the electrospun fiber mat by electrospinning, followed by the three different techniques (Electrospraying, Spraying, and Dip-Coating) for immobilizing TiO_2_ particles.

This section describes the procedure followed for each of the deposition techniques. First of all, it is important to remark that electrospinning has been used as a host matrix for the further immobilization of TiO_2_ photocatalytic particles by electrospraying, spraying, and dip-coating techniques. A schematic representation of all fabrication techniques employed for the design of the functional coatings is shown in Figure 1.

A correct dispersion of TiO_2_ particles must be obtained in the solution of these three techniques to obtain a homogeneous distribution of the particles into the functional coating. Thus, a surfactant has been employed in order to control the size and shape of the metal oxide particles. Such a surfactant is CTAB, a cationic surfactant employed in these types of particles, which favors their electrostatic stabilization [66]. The solution of TiO_2_ functional particles has the same parameters in the three deposition techniques, trying to employ similar deposition conditions for a better comparison of the three deposition techniques. An aqueous solution of TiO_2_ of 10 mM was stirred for 30 min. Then 2.5 wt% of CTAB was added to the solution in order to obtain the desired particle dispersion in the aqueous medium. This mixture was stirred for a period of time 2 h. Finally, 1 mL of 8 wt% solutions was introduced into the solution in order to improve the conductivity of the solution in the electrospraying technique by a 15 min vigorous stirring process.

#### 2.2.1. Electrospinning Process

Electrospinning was employed in order to fabricate an electrospun fiber mat (Ref sample), which acts as a polymeric matrix where the TiO_2_ particles are immobilized. According to this, an appropriate solution has been achieved by controlling parameters such as polymer concentration, molecular weight, surface tension, conductivity, and viscosity [67]. Specifically, 0.8 g of PAA and 0.128 g of β-CD were mixed in 11.6 mL of ethanol, which was used as a solvent. The solution was then stirred slowly over 24 h at room temperature in order to obtain a homogeneous mixture. After that, the solution was introduced in a syringe with a 20-gauge needle with an inner diameter of 0.6 mm and was pumped with a flow rate of 1.3 mL/h. A voltage of 15 kV was applied between the syringe tip and the sample base. The distance between both elements was 20 cm [68]. Finally, the obtained samples were thermally treated at 180 °C for 40 min in order to favor the crosslinking of the polymer to the substrate.

#### 2.2.2. Electrospraying Process

Electrospraying was conducted with the solution of TiO_2_ described above and by an Electrospinning Professional Machine (Doxa Microfluidics, Malaga, Spain). The solution was pumped through the syringe with a flow rate of 2 mL/h, and the voltage applied on the tip of the syringe was 18 kV. The distance between the nozzle and the sample was 20 cm, and the deposition was carried out for a total of 30 min (ESpr sample). The homogeneous distribution of TiO_2_ particles could be appreciated by means of the white-colored surface.

#### 2.2.3. Spraying Process

As previously mentioned, similar parameters were adjusted for the spraying process. The solution of TiO_2_ was pumped with a flow rate of 2 mL/h, with a distance of nozzle-sample of 20 cm and for about 30 min of deposition (Spr sample). The spraying was performed by an ND-SP Precision Spray Coater (Nadetech Innovations, Noáin, Spain).

#### 2.2.4. Dip-Coating Process

The dip-Coating process was carried out by ND-R Rotatory Dip-Coater (Nadetech Innovations). Electrospun fiber mat samples were immersed into the CTAB-TiO_2_ solution for a total time of 30 min. Immersion velocity was 500 mm/min, whereas the withdrawal velocity of the robot was 200 mm/min, respectively (Dip sample).

### 2.3. Morphological Characterization

Roughness and thickness characterization was obtained with confocal microscopy (model S-mart, SENSOFAR METROLOGY, Barcelona, Spain) with an objective of EPI 20×. The surface morphology of samples was studied using field emission-scanning electron microscopy (FE-SEM Hitachi S3800, Tokyo, Japan), and the particles dispersed onto the fibers can be observed and distinguished by SEM/EDX mapping.

### 2.4. Adhesion Measurements

The adhesion of the polymeric coating was analyzed in terms of the ASTM D3359 standard test method. This test consists of making X-cut penetrating the film and reaching the substrate. Then, a pressure-sensitive tape was collocated over the cut area and rapidly removed. The performance of the coating was evaluated on a scale of 0 to 5. A sharp razor blade was employed in order to trace two cuts about 40 mm long, and the angle of the intersection lines was between 30° and 45°. The tape was then collocated over the cut line, trying not to trap air bubbles under it. After 90 s of application time, the tape was rapidly removed from the surface. The X-cut area was finally inspected and rated according to the norm scale.

This paper studied the adhesion performance of samples with and without immersion in distilled water. In the case of investigating the adhesion properties of immersed samples, they were properly dried for 24 h at room temperature before performing the assay.

### 2.5. Photocatalytic Assays

#### 2.5.1. Photocatalytic Wettability

As mentioned above, one way of evaluating the photocatalytic performance of a coating is studying the wettability of the deposition while a UV light directly induces the water droplet. If the surface becomes more hydrophilic than usual, a photocatalytic performance can be considered due to the TiO_2_ nanoparticles [21]. The PAA electrospun fiber mat can also generate free radicals by the irradiation of UV light on it, so a decrease in water contact angle is presented on its surface but with lower efficiency [69]. Thus, this paper analyzes the water contact angle of the samples with and without the influence of the UV light source.

A contact angle meter (CAM 100 KSV Instruments, Burlington, VT, USA) was used in order to inspect the wettability of each sample surface. Furthermore, an optical fiber setup along with a UV light source (maximum spectra at λ = 365 nm) was employed in order to activate the TiO_2_ particles. A sessile drop was recorded in fast mode with a trigger 5 s after the drop touched the surface. After the first contact angle data, measurements of wettability were taken every 30 s, with a total amount of 20 measurements. Thus, the evolution of each contact angle has been performed during a 10 min assay in order to analyze the change of wettability on the functional surface. Water contact angles were measured using the tangent algorithm drop profile fitting method.

#### 2.5.2. Photocatalytic Activity by Dye Degradation

The other methodology to measure the photocatalytic efficiency of samples is employing an optical fiber setup, which has been described in previous works [70]. In order to conduct this assay, samples were immersed in a Rhodamine B dye solution, inking the samples with a pink-purple color, and dried for 24 h at room temperature. Afterward, the colored samples were collocated in a holder of an optical fiber setup. Visible light and UV light (maximum spectra at λ = 365 nm) sources were connected to a bifurcated fiber which was in charge of guiding both light spectrums to the holder where the sample was assembled. Subsequently, another optical fiber was used in order to direct the transmitted light to a spectrometer, which was connected to spectroscopy computer software. This assay studied the photodegradation of the organic dye colorant over time. UV light source was in charge of activating the photocatalytic response of TiO_2_ particles, whereas the visible light allowed visualizing the Rhodamine B spectrum in the computer software during the process. This assay was performed for a total of 10 h to an incident-colored area of the samples of 12.5 mm^2^.

### 2.6. Electrochemical Measurements

Electrochemical characterization was performed by potentiodynamic curves (PC) using a three-electrode cell. The coated samples were used as the working electrode; a 3 M KClsilver–silver chloride electrode (Ag/AgCl 3 M KCl) was used as the reference electrode, and a rolled platinum wire as the counter electrode. Potentiodynamic curves were obtained in triplicate (n = 3) in 0.6 M NaCl solution using a potentiostat Gamry Reference 600.

Before obtaining the PC curves, the open circuit potential (OCP) was recorded for 900 s. Subsequently, a cathodic potential step of −0.3 V (vs. OCP) was applied, and then the anodic sweep started at 0.16 mV/s until a current density limit of 0.25 mA/cm^2^ was reached.

## 3. Results and Discussion

This section is divided into five different subsections. Firstly, the morphology characterization of the samples is explained by means of SEM images, thickness, and roughness analysis. Then, the adhesion of samples with and without immersion is studied. Subsequently, photocatalytic activity is analyzed by wettability and optical fiber setup. Finally, electrochemical characterization of samples has been carried out.

### 3.1. Morphology Characterization

**Figure 2:**
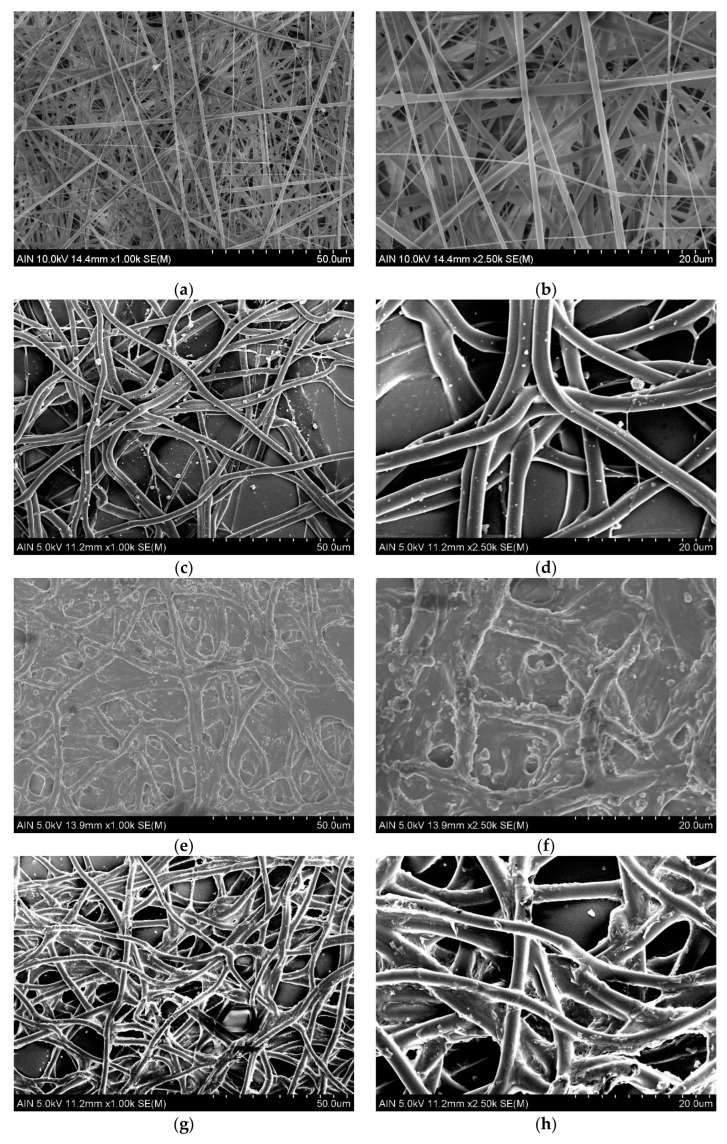
SEM images of the samples in two different magnifications: (**a**) Fiber morphology of Ref sample with ×1000 magnification; (**b**) Fiber morphology of Ref sample with ×2500 magnification; (**c**) Fiber and TiO_2_ nanoparticles morphology of ESpr sample with ×1000 magnification; (**d**) Fiber and TiO_2_ nanoparticles morphology of ESpr sample with ×2500 magnification; (**e**) Fiber and TiO_2_ nanoparticles morphology of Spr sample with ×1000 magnification; (**f**) Fiber and TiO_2_ nanoparticles morphology of Spr sample with ×2500 magnification; (**g**) Fiber and TiO_2_ nanoparticles morphology of Dip sample with ×1000 magnification; (**h**) Fiber and TiO_2_ nanoparticles morphology of Dip sample with ×2500 magnification.

The resultant SEM images of all the analyzed samples in this study are collected in Figure 2. Polymeric fibers can be distinguished in Figure 2b, obtained by the electrospinning process. A homogeneous distribution of fibers has been achieved through the coating, having similar fiber diameter values. This electrospun fiber matrix was then used as a base element for the TiO_2_ nanoparticles. Figure 2d shows the immobilization of TiO_2_ nanoparticles into the electrospun fiber mat by the electrospraying technique. As can be appreciated in the images, this deposition process achieves well-dispersed spherical nanoparticles adhered to the fibers. In the case of spraying deposition, which is illustrated in Figure 2f, a bigger amount of TiO_2_ particles has been immobilized with the same deposition condition. The particles form an agglomeration among each other and notoriously increase the diameter of the electrospun fibers. Finally, the result of the dip-coating deposition procedure is represented in Figure 2h, and a correct immobilization of TiO_2_ nanoparticles through the fibers can be observed. However, the shapes of the nanoparticles were not homogeneous among each other, and the distribution of differently shaped nanoparticles was dispersed through the electrospun fiber mat. Thus, the three deposition techniques allow for obtaining different configurations of TiO_2_ nanoparticles over the immobilization polymeric matrix.

**Figure 3:**
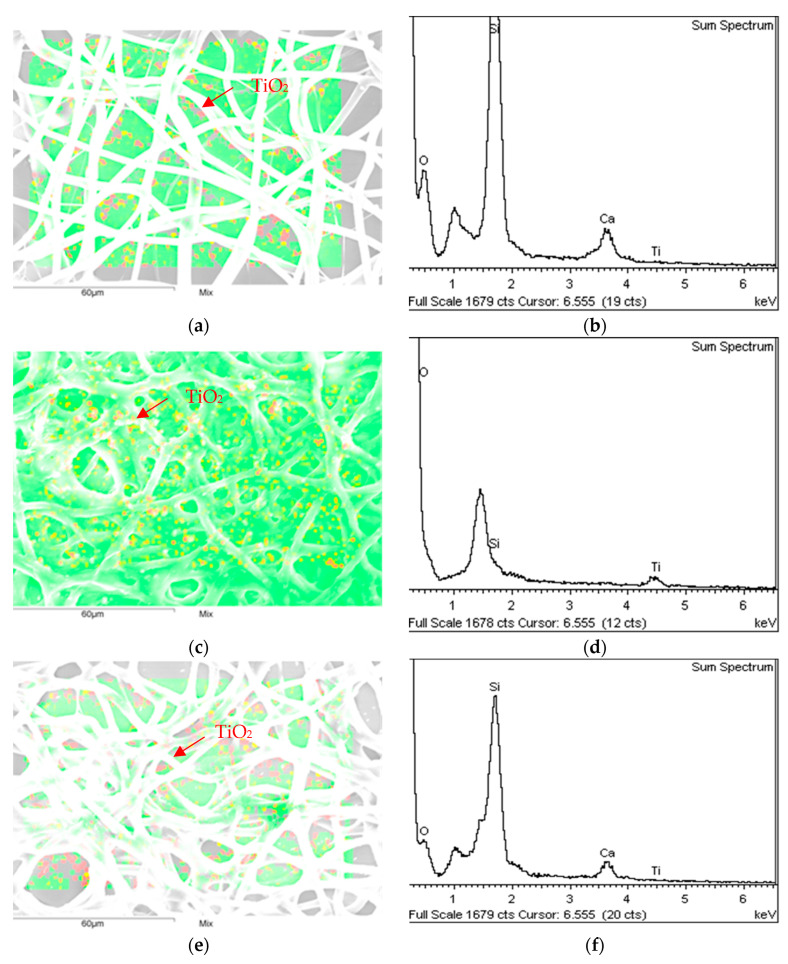
EDX graphs and 2D mapping for the analyzed samples: (**a**) TiO_2_ nanoparticles shown by a 2D mapping of the ESpr sample; (**b**) Elements peaks in EDX graph of the ESpr sample; (**c**) TiO_2_ nanoparticles shown by a 2D mapping of the Spr sample; (**d**) Elements peaks in EDX graph of the Spr sample; (**e**) TiO_2_ nanoparticles shown by a 2D mapping of the Dip sample; (**f**) Elements peaks in EDX graph of the Spr sample.

With the aim of confirming the presence of TiO_2_, EDX mapping images were taken for the three samples. Figure 3a,c,e show the presence of photocatalytic particles along the surface. Thus, the particles are dispersed along the coating in the three cases. The EDX graphic peaks show the presence of Ti, being more noticeable in the spraying sample.

**Figure 4:**
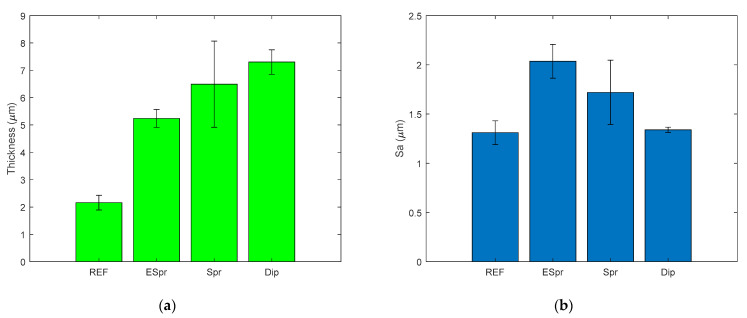
Roughness and thickness results of the analyzed functional coatings: (**a**) Thickness bar graph of the Ref, ESpr, Spr, and Dip samples with their corresponding value of standard deviation; (**b**) Roughness bar graph of the Ref, ESpr, Spr, and Dip samples with their corresponding value of standard deviation.

Using confocal microscopy, a thickness parameter and a roughness parameter such as arithmetical mean height (Sa) have been studied. The results are shown in Figure 4. A notorious increment of thickness can be appreciated among Ref and the TiO_2_ samples in Figure 4a due to the deposition of these photocatalytic particles along the surface. ESpr and Dip samples present an almost homogeneous thickness in the coating. However, the Spr sample exhibits a wider dispersion of coating thickness as a consequence of the particle agglomerations. The ESpr specimen has a lower thickness because of the size and shape of the TiO_2_ particles distributed along the electrospun matrix. Thus, the Dip sample has a greater thickness due to the difference in particle sizes and shapes.

The roughness of the samples was remarkably similar. Agglomerations in the Spr sample give rise to a lower roughness because of the fiber diameter increment. Thus, the porosity of the fibers is reduced. The amount of TiO_2_ particles present in the Dip sample is lower than the two others, but the roughness of this sample is so similar to the reference electrospun matrix.

### 3.2. Adhesion Measurements

In order to analyze the interaction between substrate and coating, ASTM D3359 Method A Rating standardization has been followed for sample characterizations, with and without the immersion of the specimens in distilled water. As previously mentioned, the base matrix was composed of PAA and β-CD, which was in charge of crosslinking the polymeric coating to the substrate after the heat treatment [71]. Once this polymeric base was deposited, the other deposition techniques were carried out without further treatments.

**Figure 5:**
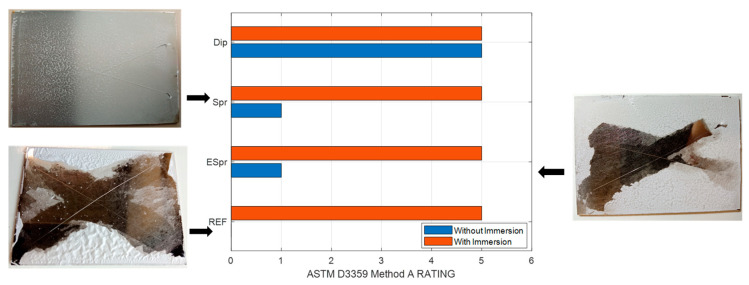
ASTM D3359 Method A Rating standardization adhesion results of functional coatings with and without distilled water immersion treatment. Assay picture results of Ref without immersion, ESpr without immersion, and Spr with immersion.

In the case of samples without immersion, the electrospun fiber mat composed of PAA+ β-CD (Ref) could not support the test, and the coating was removed from the substrate (rating value of 0), as shown in Figure 5. ESpr and Spr samples also showed a low adhesion property because almost all the crossed area was removed (rating value of 1). Finally, the Dip sample obtained the maximum rating (rating value of 5) because the coating remained completely adhered to the substrate. In the case of samples with immersion, an enhancement in the adhesion of samples was obtained, achieving in all specimens the maximum grade of adhesion by this standard methodology. The increase in adhesion by this immersion treatment is due to the high density of carboxylic acid groups and negative charges of the PAA weak polyelectrolyte, which give rise to both electrostatic and Van der Waals complex balance interactions promoting a strong immobilization into the substrate [72]. This concludes that submerged samples exhibit excellent adhesion behavior according to ASTM D3359 Method A Rating [68].

### 3.3. Photocatalytic Assays

#### 3.3.1. Photocatalytic Wettability

**Figure 6:**
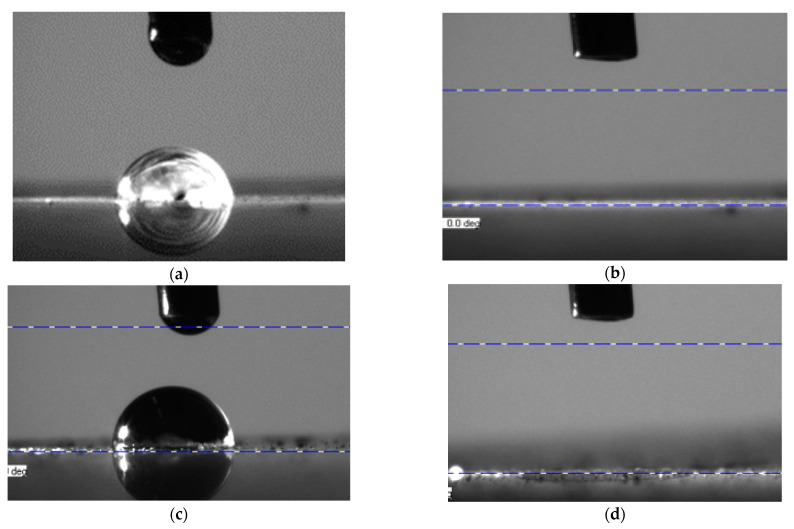
Photocatalytic wettability test results for ESpr and Spr samples: (**a**) Beginning of the assay, the initial contact angle of the water drop in the ESpr sample; (**b**) End of the assay, the final contact angle of the water drop in ESpr sample after irradiating with UV light; (**c**) Beginning of the assay, the initial contact angle of the water drop in Spr sample; (**d**) End of the assay, the final contact angle of the water drop in Spr sample after irradiating with UV light.

As previously mentioned, the photocatalytic response of samples has also been measured by means of wettability response under a UV light incidence in the area where the water drop was collocated. If the coating presents a super-hydrophilic response to the UV light beam, a photocatalytic reaction can be considered [21]. The change in wettability of ESpr and Spr samples under UV light irradiance can be appreciated in Figure 6, from a hydrophilic state to a complete disappearance of the water drop during the assay because of the super-hydrophilic behavior.

**Figure 7:**
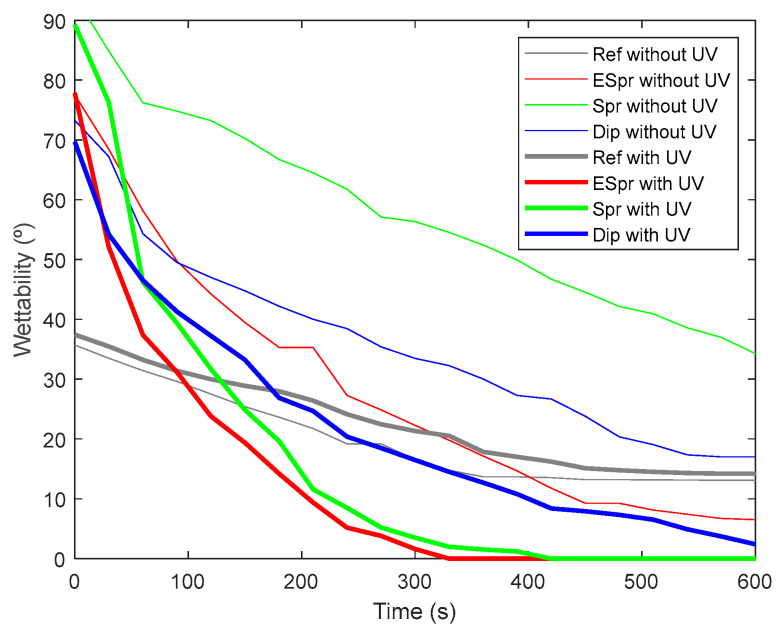
Water drop contact angle decreases results with and without UV incidence to the functional area of the water drop, along a total time of 10 min.

A rapid change from the hydrophilic to the super-hydrophilic state of two samples (ESpr, Spr, and Dip) can be concluded in Figure 7. ESpr started with an initial water drop contact angle of 78°, Spr with an initial angle of 90°, and Dip with 70° of contact angle. The water drop situated in the ESpr specimen did not achieve the total disappearance in the 10 min of assay duration without using UV light. Nevertheless, with the photocatalytic activation of UV irradiance, the disappearance of the water drop was achieved in only 5 min. Regarding the Spr sample, the difference between employing UV and not was greater if compared with the ESpr sample. The overall disappearance of the water drop took almost 6 min, whereas the case of not irradiating the sample finished with a degree of 45° of contact angle. The dip sample presents a lower photocatalytic wettability activation, but the phenomenon can also be distinguished. This sample needed 10 min to eliminate the water drop totally, and without the photocatalytic activation, the water drop finished with a contact angle of 18°. In order to corroborate that the super-hydrophilicity was achieved thanks to the TiO_2_ particles and not by using simply UV light, the same assay was performed on the Ref sample. Starting with a contact angle of 37°, the final contact angle in both cases (with and without UV irradiation) was less than 15°. As previously mentioned, free radicals were also formed in the PAA base matrix due to the UV light, but in this case, no contact angle alteration can be observed. Thus, the UV light beam had no impact on this sample’s wettability.

#### 3.3.2. Photocatalytic Activity by Dye Degradation

**Figure 8:**
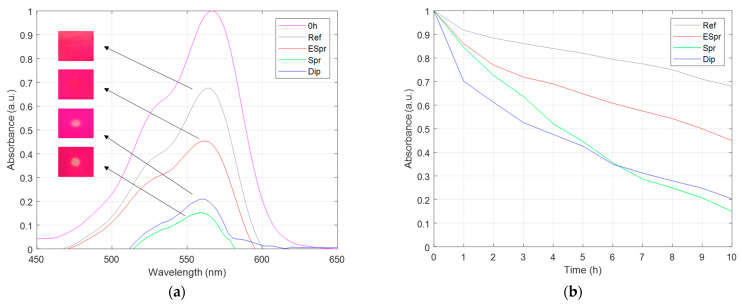
Photocatalytic degradation of RhB organic dye under UV light irradiation after depositing it into the functional coatings: (**a**) Final spectrums of dye degradation of all samples for a 10 h photocatalytic test; (**b**) RhB maximum peak decrease along time for each sample.

Another methodology to characterize the photocatalytic response of coatings is studying the photodegradation of a dye by employing the previously described optical fiber setup [70,73]. Typical peaks of this colorant are shown in Figure 8, with a lower peak centered at 525 nm and the main peak centered at 570 nm. After 10 h of UV irradiance to the organic dye, the sample without any TiO_2_ photocatalytic particles in it presented a degradation of 32%. Although the coating was not photocatalytic, UV light has the capacity to form free radicals in the PAA base matrix, achieving a small degradation of the dye due to the interaction of these radicals with the dye [69]. The ESpr specimen achieved a discoloration of 55%, being the worst response among the three photocatalytic samples. The Spr and Dip samples reach 85% and 80% degradation, respectively. Spr had the greater response for photodegrading the organic dye, which makes sense because it was the sample with the greater amount of particles. According to the bibliography, particle size and shape also play an important role in photocatalysis, as mentioned in the previous section [27]. However, this fact could not be corroborated by this assay. The degradation of the dye during the test is represented in Figure 8b. The Dip sample had the greater degradation rate in the initial hours of the assay, but then the graph was smooth. The Spr sample had a constant decrement of peak absorbance, achieving greater discoloration of the dye.

### 3.4. Electrochemical Evaluation

**Figure 9:**
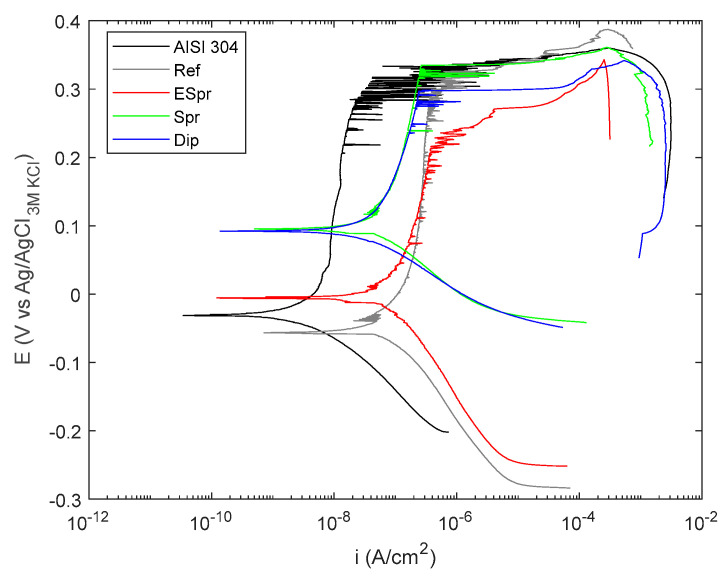
Electrochemical curve results of the potentiodynamic evaluation of functional coatings deposited into AISI304 substrates.

The representative polarization curves corresponding to each coating system and the bare stainless steel (AISI 304) are shown in Figure 9. As can be observed, the reference coating (Ref) shifts its vertical anodic branch towards higher current density values regarding AISI304 but keeps a similar pitting potential (350 mV). Such higher passive current density values result from the permeability of the PAA, which absorbs the electrolyte, likely increasing the area exposed to the electrolyte and, therefore, the current flow. Similar passive current density values are depicted by the coating containing TiO_2_ deposited by electrospraying (ESpr). Conversely, this coating shows lower localized corrosion resistance since the pitting potential decreases by nearly 100 mV compared to the reference coating (ref) and the bare stainless steel (AISI304).

On the other hand, the TiO_2_ coatings deposited by spraying (Spr) and dipping (Dip) show slightly lower passive current densities compared to Ref and ESpr coatings and the same pitting potential as the bare stainless steel. Conversely, the corrosion potential shifts about ~100 mV in an anodic sense towards nobler values. The changes experienced by the potentiodynamic curves result in an increase in the coating thickness.

The deposition of these coatings on stainless steel provides photocatalytic functionality to the surface of the stainless steel. It is important to highlight that despite the coatings not providing additional corrosion protection, in most cases, the coating deposition does not have detrimental effects on corrosion resistance since both corrosion kinetics and corrosion susceptibility to localized attack range in the same order as the bare stainless steel 304L.

## 4. Discussion

**Figure 10:**
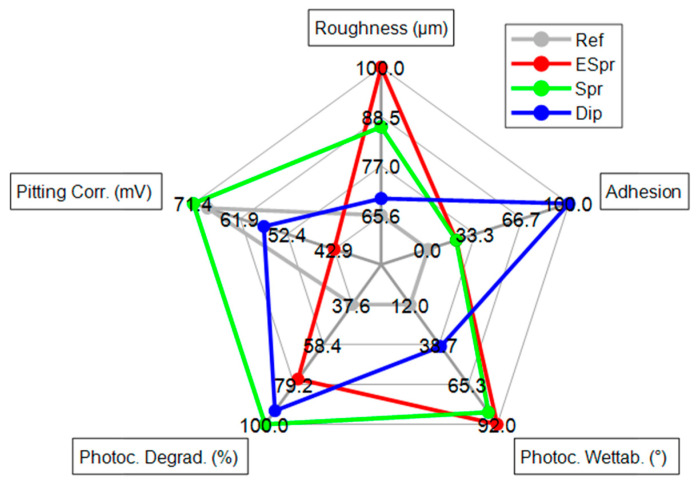
Five of the main features analyzed in this study resumed in a spider diagram for all photocatalytic samples on a scale from 0 to 100.

A summary diagram comparison of the results achieved by this study has been elaborated (Figure 10). Results are normalized into a scale from 0 to 100 in order to rate each sample. The roughness feature expresses the Sa value of the samples. The ESpr sample has the greatest roughness value and, thus, a greater functional area for photocatalytic reactions. Adhesion of specimens is rated from 0–5 using the ASTM D3359 Method A Rating scale. Photocatalytic wettability is expressed in Figure 10 by means of the angle decrease by the water drop in 300s of UV light exposure. Photocatalytic degradation of the dye is represented in the diagram by the percentage of degradation of the dye in each case. Finally, the electrochemical analysis is represented by means of pitting corrosion of each sample. With greater values of pitting potentials, a better performance is obtained. By this comparison, it can be appreciated how the spraying specimen is the most effective sample, taking into account all the analyzed features.

## 5. Conclusions

This work has been capable of comparing three different deposition techniques of TiO_2_ nanoparticles for photocatalytic applications. It has been demonstrated that the size, shape, and total amount of TiO_2_ particles in the outer surface of the samples have played a key role in this study. The electrospraying technique has achieved a great distribution of TiO_2_ spherical nanoparticles, which are perfectly adhered through the electrospun fibers without any signal of aggregation. However, the spraying technique has shown the opposite effect because the formation of agglomeration of them has been observed along the electrospun fiber matrix. In the case of the dip-coating technique, variable shapes and sizes of particles were obtained, although a good dispersion of TiO_2_ nanoparticles onto the electrospun fibers was observed. In the case of photocatalytic wettability, electrospraying and spraying techniques have presented the best results, with the complete vanishing of the water drop in less than 5 min of UV irradiation. On the other hand, the results provided by the photocatalytic degradation test conclude that spraying and dip-coating techniques present the best behavior, reaching 85% and 80% dye degradation in 10 h. Finally, according to the experimental results, the sample achieved by the spraying deposition process is the most effective regarding both assays, and the amount of TiO_2_ particles in the coating has a more important role in comparison with good dispersion and correct control over particle size and shape.

## Data Availability

Not applicable.
